# Pre-copulatory reproductive behaviours are preserved in *Drosophila melanogaster* infected with bacteria

**DOI:** 10.1098/rspb.2022.0492

**Published:** 2022-05-11

**Authors:** Saloni Rose, Esteban J. Beckwith, Charlotte Burmester, Robin C. May, Marc S. Dionne, Carolina Rezaval

**Affiliations:** ^1^ School of Biosciences, University of Birmingham, Birmingham B15 2TT, UK; ^2^ Institute of Microbiology and Infection, University of Birmingham, Birmingham B15 2TT, UK; ^3^ MRC Centre for Molecular Bacteriology and Infection and Department of Life Sciences, Imperial College London, London SW7 2AZ, UK; ^4^ Instituto de Fisiología, Biología Molecular y Neurociencias (IFIBYNE), UBA-CONICET, Buenos Aires, Argentina

**Keywords:** reproductive behaviours, reproduction and immunity trade-off, bacterial infection, *Drosophila*, courtship

## Abstract

The activation of the immune system upon infection exerts a huge energetic demand on an individual, likely decreasing available resources for other vital processes, like reproduction. The factors that determine the trade-off between defensive and reproductive traits remain poorly understood. Here, we exploit the experimental tractability of the fruit fly *Drosophila melanogaster* to systematically assess the impact of immune system activation on pre-copulatory reproductive behaviour. Contrary to expectations, we found that male flies undergoing an immune activation continue to display high levels of courtship and mating success. Similarly, immune-challenged female flies remain highly sexually receptive. By combining behavioural paradigms, a diverse panel of pathogens and genetic strategies to induce the fly immune system, we show that pre-copulatory reproductive behaviours are preserved in infected flies, despite the significant metabolic cost of infection.

## 1. Introduction

Life-history theory argues that there is a trade-off between energetically expensive traits, like reproduction and immunity [1]. Animals expend a considerable amount of energy in pre-copulatory traits, such as courtship behaviours to attract a potential mate or post-copulatory traits, like producing eggs [1]. At the same time, when challenged with an infection, individuals must allocate resources to mount an effective immune response and increase the chances of survival [2,3]. How do individuals prioritize and balance their investment in reproduction and immune defence? Several studies have shown that individuals exposed to harmful infections prioritize defence over reproductive strategies [4,5]. For instance, upon infection, some insects and birds show decreased egg production [6], whereas infected crickets and fish show reduced sperm production and viability [7]. Further, time invested in courtship and overall performance is affected in response to infection in birds and fish [8,9]. On the other hand, studies in several species have indicated increased reproductive effort during infection [10,11]. The selection between investment strategies is thought to depend on the host's internal conditions (e.g. age and genetic background) [12,13] and extrinsic factors (e.g. nature of the infection, pathogen's virulence and its relationship with the host) [14]. Therefore, investigating how the infection type, dose, timing and virulence modulate reproductive traits is essential for understanding variation in reproductive effort across species.

*Drosophila melanogaster* is a powerful model organism to study the interaction between reproduction and immunity. First, the courtship ritual in flies is composed of complex innate behaviours that culminate with copulation and has been studied for more than 100 years [15]. To woo a female and assess her suitability, male flies perform a sequence of stereotyped courtship steps that allow the exchange of sensory cues. Females show acceptance or rejection behaviours in response to the male's courtship display [15]. Second, *Drosophila* has a well-characterized immune system [16]. While a hallmark of the immune activation is the synthesis of antimicrobial peptides (AMPs), it is characterized by a marked transcriptomic switch with a profound metabolic impact [17]. These changes take place primarily in the fat body and are under the control of two different cascades: Toll and Imd pathways, which activate different NF-κB-like factors and induce the innate immune response [16]. Notably, many aspects of innate immunity are conserved between flies and mammals, such as NF-κB family transcription factors and signal transduction pathways, and the organization of the immune system is highly analogous [16]. A few studies have investigated the impact of pathogen infection on female reproductive traits, like oviposition [18], and some aspects of male courtship [19]. However, how infection modulates reproductive behaviours in *Drosophila* remains poorly understood.

Here, we systematically assess the impact of infections with pathogenic and non-pathogenic bacterial species on male courtship behaviours and female sexual receptivity in *D. melanogaster*. We evaluate bacterial strains that are phylogenetically diverse, have different virulence and host–pathogen relationships and impose differing fitness costs on flies [20–23]. Our findings indicate that male courtship behaviours, female sexual receptivity and mating success are safeguarded during bacterial infections. Moreover, reproductive behaviours remain unaltered upon genetic activation of Toll and Imd immune pathways. Our study demonstrates that pre-copulatory reproductive behaviours remain preserved in infected flies despite the significant metabolic cost of infection.

## 2. Material and methods

Also see extended methods.

### (a) *Drosophila* stocks

Wild-type *D. melanogaster* lines used in the study include Canton-S and Dahomey strains. Transgenic lines include C564-GAL4 (BDSC 6982), UAS-Toll10B (BDSC 58987) and UAS-Imd (gift from Markus Knaden). Flies were raised on a standard cornmeal at 25°C, 50–60% humidity with a 12 h light/dark cycle. Virgin male and female flies were aged for 5–7 days in same-sex groups of 15–20 before experimentation.

### (b) Bacterial infection

The bacterial strains used in this study include *Serratia marcescens* (*DB11*), *Staphylococcus aureus* (*SH1000*), *Listeria monocytogenes* (*EGD-e*), *Escherichia coli* (*DH5α*), *Pectinobacterium carotovorum carotovorum 15* (*ECC15*) and *Micrococcus luteus* (clinical isolate, gift from Prof. William Wade, King's College London). The bacterial strains were cultured overnight (see extended methods) at and cultures were pelleted by centrifugation at 4500*g* for 2 min. The pellet was diluted in filter-sterilized phosphate-buffered saline (PBS) to a defined concentration. Fifty nanolitres of the diluted bacterial solution was injected employing a nano-injector (MPP1-3 Pressure Injector, Applied Scientific Instrumentation) into the abdomen of anaesthetized flies as per [24].

### (c) Survival assay

Infected flies and controls were placed in groups of 10–15 in.vials at 29°C. The number of live flies infected with pathogenic strains was counted at regular intervals until all the infected flies were dead. Flies injected with non-pathogenic strains were counted at regular intervals for 72 h.

### (d) Behavioural assays and parameters

All behavioural experiments were done in between zeitgeber time (ZT01) and (ZT10) at 25°C. Mating assays were carried out in courtship chambers (20 mm in diameter and 5 mm in height), which have built-in dividers that allow separation of the flies before the experiment. For single pair mating assay*,* flies were injected with bacteria or vehicle solution (PBS) and immediately placed in the courtship chamber with food. Before the behavioural measurement began, the uninfected flies of the opposite sex were introduced using a fly aspirator. The dividers were opened before the assay and behaviours were recorded for 1 h. For mate choice assays*,* a focal fly was given a choice between an infected and a healthy (PBS) mate. The infected and healthy flies were marked with acrylic paint 48 h before experimentation. After injection, both infected and PBS flies were transferred to a courtship chamber with food. The focal fly was aspirated into the chamber before behavioural experimentation and behaviours were recorded for 1 h. Courtship index (in experiments with infected males and controls) was measured as the proportion of time the male spends courting from the beginning of courtship until 10 min or end of copulation. Mating success was measured as the percentage of flies that mated within 1 h. Copulation latency (in experiments with infected females and controls) was measured as the time taken to copulate from the start of courtship. For competitive mating assays, the focal fly's first mate choice was recorded (i.e. if the fly chose to mate with a healthy or infected mate).

### (e) Statistical analysis

All statistical analyses and data visualization (ggplot2, R Markdown) were performed using R studio (R v. 3.6). We used mixed effect regression models to explore the effect of infection timing and concentration on male courtship behaviours. Kruskal–Wallis followed by Dunn's test with Bonferroni corrections were used for *post hoc* comparisons across different treatments. Fisher's exact test was used to analyse count data and log-rank test for survival data. Differences were accepted as significant at *p* < 0.05. Statistical analysis and sample sizes are summarized in the electronic supplementary material.

## 3. Results

### (a) Male courtship behaviour is maintained during infections with non-pathogenic bacteria

To reliably produce infection phenotypes and assess the consequences in behaviour, we used a nano-injector to deliver precise volumes of bacterial solution into the abdomen of wild-type Canton S (CS) flies [24]. As a starting point, we chose three different non-pathogenic bacteria: *ECC15*, *E. coli* and *M. luteus,* which activate the fly's innate immune system without affecting lifespan [21,25,26]. Gram-negative bacteria *ECC15* and *E. coli* induce the Imd pathway, while the Gram-positive bacterium *M. luteus* activates the Toll pathway [16]. As expected, CS males infected with either ECC15, *E. coli* or *M. luteus* showed a survival rate similar to that of uninfected and sham-infected flies (electronic supplementary material, figure S1A–C). Given that flies rapidly clear non-pathogenic bacteria from their bodies [21,27], we measured behaviour 5–6 h post-infection, when the bacterial load is still detectable and the immune activation is robust.

**Figure 1.**
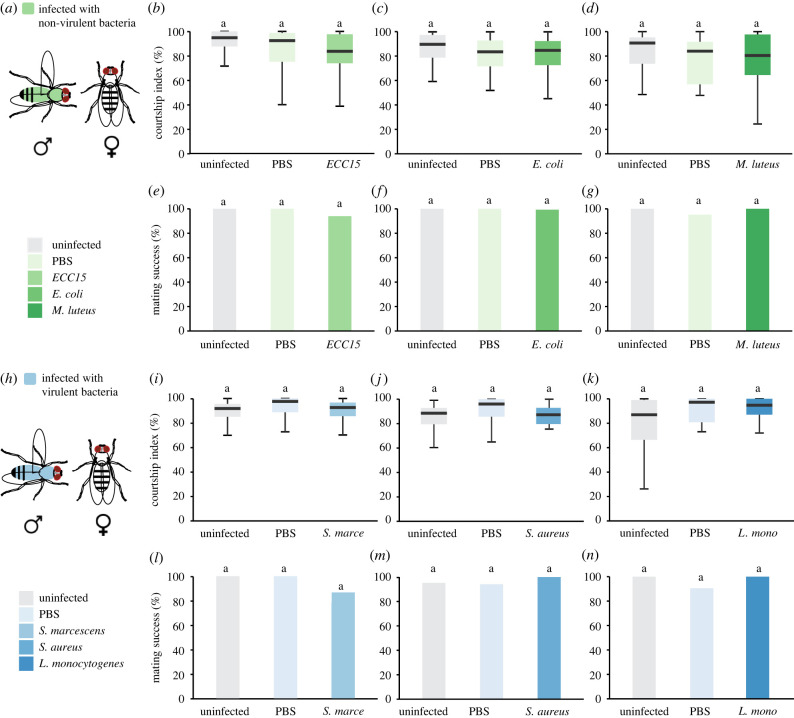
Effect of bacterial infections on male courtship behaviour. (*a,h*) Male CS flies were injected with three non-pathogenic (*a*) and pathogenic (*h*) strains and tested in a single pair courtship assay with an uninfected virgin female. (*b*) Courtship index and (*e*) mating success of males infected with *ECC15* and their respective controls (*n* = 31–32). (*c*) Courtship index and (*f*) mating success of males infected with *E. coli* and their respective controls (*n* = 22–31). (*d*) Courtship index and (*g*) mating success of males infected with *M. luteus* and their respective controls (*n* = 21–22). (*i*) Courtship index and (*l*) mating success of males infected with *S. marcescens* and their respective controls (*n* = 29–30). (*j*) Courtship index and (*m*) mating success of males infected with *S. aureus* and their respective controls (*n* = 17–21). (*k*) Courtship index and (*n*) mating success of males infected with *L. monocytogenes* and their respective controls (*n* = 20–23). Dunn's test in (*b*–*d*), (*i*–*k*) and Fisher's test in (*e*–*g*), (*l*–*n*). Courtship indices and mating success are presented as percentage. A detailed description of the statistics employed can be found in the electronic supplementary material. (Online version in colour.)

To assess the effects of non-pathogenic bacteria on male courtship, we performed single mating assays, where an infected male was paired with a healthy virgin female (figure 1*a*). As expected, uninfected and sham-infected males (PBS) spent most of the time courting the female. Interestingly, males infected with either ECC15, *E. coli* or *M. luteus* displayed comparable courtship levels to that of controls (figure 1*b–d*). Most control males successfully copulated within 1 h and infected males showed a similar rate of copulation (figure 1*e–g*). To further explore the trade-off between male courtship behaviours and immune activation, we evaluated the behaviour of ECC15 infected flies at three different time points (3, 5 and 8 h) and doses (OD_600_ = 0.5, 1 and 2) (electronic supplementary material, figure S2). Earlier or later time points, as well as changes in the bacterial load, did not reveal a change in male courtship behaviour or mating success (electronic supplementary material, figure S2).

Next, we asked if the lack of effect of infection on male courtship could be generalized to a second *Drosophila* wild-type strain. To this end, we selected Dahomey, an outbred strain isolated from Benin [28]. We found that, like CS, Dahomey males did not alter their courtship behaviour in response to infection (electronic supplementary material, figure S3). These results suggest that male courtship behaviours are maintained during the response to non-pathogenic infections.

### (b) Wild-type males sustain courtship behaviour during pathogenic and lethal infections

Our findings show that male courtship behaviours are not affected by infections with non-pathogenic strains. We reasoned that more virulent and pathogenic bacteria that can grow within the flies may have a stronger impact on male pre-copulatory behaviours. To test this hypothesis, we chose three pathogenic bacterial species that have been shown to negatively affect *Drosophila* physiology and induce lethality: *S. marcescens, S. aureus* and *L. monocytogenes* (figure 1*h*). Infecting male flies with the natural insect pathogen *S. marcescens* caused 100% mortality within 9 h upon injection (electronic supplementary material, figure S1D). By contrast, uninfected flies and sham-infected flies survived through the observation time. Non-natural pathogens for flies, such as *S. aureus* or *L. monocytogenes*, induced lethality within 24 h (electronic supplementary material, figure S1E) and approximately 7 days (electronic supplementary material, figure S1F), respectively, as previously reported [20,21,23].

Considering the survival data, we performed behavioural experiments with *S. marcescens, S. aureus* and *L. monocytogenes* at 5–6 h, 8–9 h and 24–25 h post-infection, respectively, when the immune response is mounted, and the infections are advanced but not too detrimental for the flies (e.g. locomotion abilities remain intact; electronic supplementary material, figure S4). Surprisingly, none of these lethal infections altered male courtship behaviour. There were no significant changes in the courtship index of infected males with either *S. marcescens* (figure 1*i*), *S. aureus* (figure 1*j*) or *L. monocytogenes* (figure 1*k*). Further, these lethal infections did not compromise male mating success (figure 1*l–n*). To investigate if additional infection timings might reveal a trade-off between defensive and pre-copulatory behaviours, we tested *S. marcescens* infected males at earlier (4–5 h) and later (6–7 h) time points. While we did not find changes 4 h post-infection, we detected a significant decrease in courtship index and mating success 6 h post-infection in infected males (electronic supplementary material, figure S5B,C). However, these flies were lethargic and presented a drastic reduction of locomotor activity (electronic supplementary material, figure S5D–F), arguing for an unspecific effect on pre-copulatory behaviours. Selection might favour flies that do not increase immune investment as there is no chance to overcome the infection. We thus asked if lower doses of S. *marcescens* would reveal an effect of infection on courtship. We thus tested two more infections doses of this bacteria (OD_600_ = 0.01 and OD_600_ = 0.001). While the lower doses extended lifespan (electronic supplementary material, figure S6A), there was still no effect on pre-copulatory behaviours (electronic supplementary material, figure S6C,D). Finally, the courtship behaviours of Dahomey male flies infected with any of these pathogenic bacterial strains remained unaffected (electronic supplementary material, figure S3).

Altogether, male courtship behaviours seem unaltered during bacterial infections, even in the face of a life-threatening situation. We reasoned that the high level of sex drive observed in control flies (figure 1) might mask the modulatory effect of immune activation. To decrease basal courtship levels, we presented CS males with mated females, which are reluctant to copulate and are therefore unattractive courtship targets [29]. As expected, uninfected CS males showed decreased courtship index towards mated females (approx. 20%). However, when presented with mated females, male flies infected with either pathogenic (*S. marcescens*) or non-pathogenic (*ECC15*, *M. luteus*) bacteria exhibited similar courtship behaviour to that of controls (PBS or uninjected) (electronic supplementary material, table S1).

From our experiments, we conclude that CS and Dahomey males infected with either pathogenic or non-pathogenic strains maintain their courtship efforts while they mount a costly immune response.

### (c) Female flies sustain their sexual receptivity during bacterial infections

**Figure 2.**
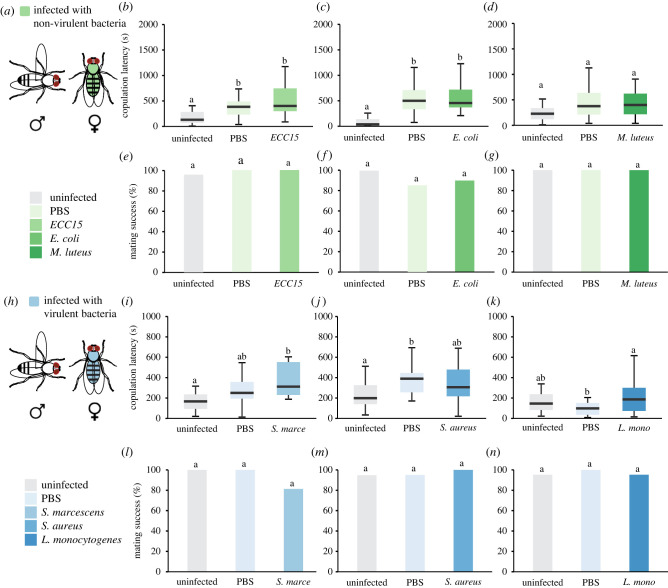
Effect of bacterial infections on female sexual receptivity. (*a,h*) Virgin female CS flies were injected with three different non-pathogenic (*a*) and pathogenic (*h*) strains and tested in a single pair courtship assay with an uninfected male. (*b*) Copulation latency and (*e*) mating success of females infected with *ECC15* and their respective controls (*n* = 20–24). (*c*) Copulation latency and (*f*) mating success of females infected with *E. coli* and their respective controls (*n* = 19–20). (*d*) Copulation latency and (*g*) mating success of males infected with *M. luteus* and their respective controls (*n* = 31–32). (*i*) Copulation latency and (*l*) mating success of females infected with *S. marcescens* and their respective controls (*n* = 16–19). (*j*) Copulation latency and (*m*) mating success of females infected with *S. aureus* and their respective controls (*n* = 19–20). (*k*) Copulation latency and (*n*) mating success of females infected with *L. monocytogenes* and their respective controls (*n* = 20–21). Dunn's test in (*b*–*d*), (*i*–*j*) and Fisher's test in (*e*–*g*), (*l*–*n*). Copulation latency is measured in seconds and mating success is presented as percentage. A detailed description of the statistics employed can be found in the electronic supplementary material. (Online version in colour.)

In many animals, including flies, there is sexual dimorphism in survival, pathology, bacterial load and activity in response to infections [30,31]. In addition, the costs of reproduction might be different between sexes. Hence, we asked if female reproduction behaviour is differentially modulated by infections. We injected CS and Dahomey virgin females with the same non-pathogenic (figure 2*a*) and pathogenic (figure 2*h*) bacteria we employed for males and measured copulation latency and mating success, both of which are proxies for female receptivity. For the non-pathogenic bacteria, we found that both infected and sham-infected females exhibit a higher latency to copulation than uninfected controls. However, there were no differences between sham-infected and infected females, indicating that bacterial infection itself does not influence copulation latency (figure 2*b–d*). Similarly, female mating success remained unchanged upon infection with non-pathogenic bacteria (figure 2*e–g*). The pathogenic strains dramatically reduced female survival (electronic supplementary material, figure S1D–F); however, these infections did not affect the latency to copulation in females (figure 2*i–k*). In addition, female mating success remained unaffected upon pathogenic infection (figure 2*l–n*). Dahomey virgin females infected with all these bacteria strains showed a similar trend (electronic supplementary material, figure S7). We therefore conclude that *Drosophila* female receptivity is not affected during the response to infections.

### (d) Effect of social context on pre-copulatory behaviours under infection conditions

**Figure 3.**
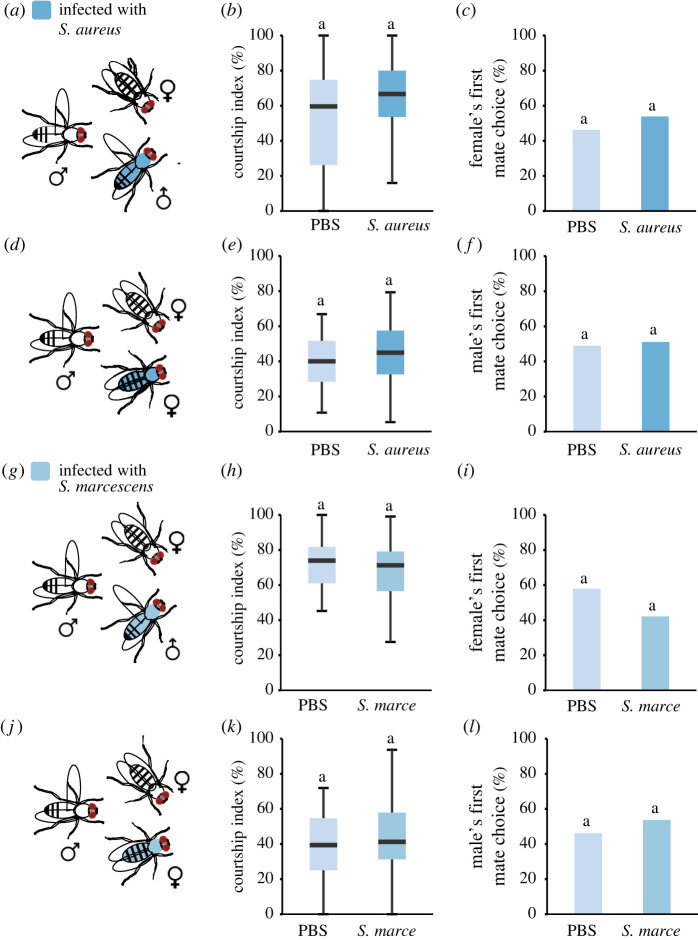
Effect of pathogenic infections on courtship behaviours and mate selection in a choice context. (*a*) A focal female was given a choice between a male injected with PBS and a male infected with *S. aureus*. (*b*) Courtship index of the PBS or infected male towards the focal female. (*c*) Female's first mate choice (*n* = 57). (*d*) A focal male was given a choice between a PBS female and a female infected with *S. aureus*. (*e*) Courtship index of the focal male. (*f*) Male's first mate choice (*n* = 45). (*g*) A focal female was given a choice between a PBS male and a male infected with *S. marcescens*. (*h*) Courtship index of the PBS or infected male. (*i*) Female's first mate choice (*n* = 38). (*j*) A focal male was given a choice between a PBS female and a female infected with *S. marcescens*. (*k*) Courtship index of the focal male towards PBS or infected female. (*l*) Male's first mate choice (*n* = 54). Wilcoxon's test in (*b*), (*e*), (*h*) and (*k*) and Fisher's test in (*c*), (*f*), (*i*) and (*l*). Courtship indices and first choice are presented as percentage. A detailed description of the statistics employed can be found in the electronic supplementary material. (Online version in colour.)

Male reproductive behaviours are modulated by social context [32]. We thus wondered whether infected male flies would behave differently in the presence of a healthy male competitor. To test this, we paired a CS virgin female with a healthy male and a male infected with *S. aureus* (figure 3*a*). Interestingly, we found that infected males with this pathogenic strain courted to the same extent as the uninfected male (figure 3*b*). In addition, healthy females did not preferentially mate with healthy males over infected males (figure 3*c*). We similarly tested whether healthy male flies would behave differently towards healthy or infected females (figure 3*d*). When given a simultaneous choice between *S. aureus*-infected females and sham-infected females, these males spent a similar amount of time courting each female in a competitive assay (figure 3*e*). Moreover, they mated equally with healthy or *S. aureus-*infected females (figure 3*f*). Extending these studies to a second pathogenic strain, *S. marcescens,* showed an identical pattern (figure 3*g–l*). These results suggest that a more complex social context does not change the reproductive performance of infected flies.

### (e) Genetic activation of the immune pathways has limited impact on male courtship behaviour and female sexual receptivity

**Figure 4.**
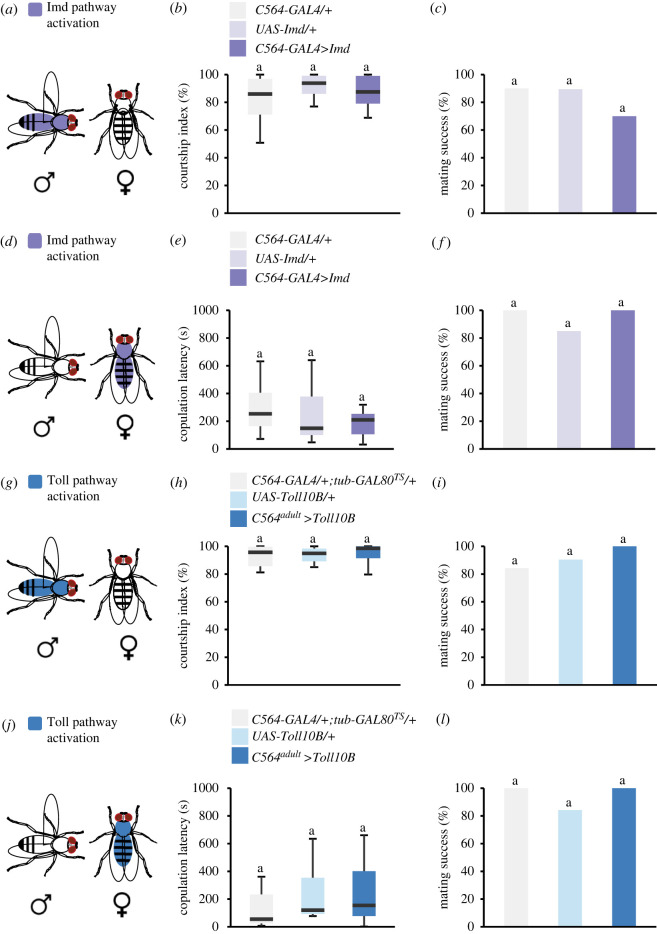
Effect of immune system activation on male courtship behaviour. (*a,d*) The Imd pathway was artificially activated in the fat body of male or virgin female flies. (*b–c*) Courtship index and mating success of *C564-GAL4 > UAS-Imd* male flies (*n* = 19–20). (*e,f*) Copulation latency and mating success of *C564-GAL4 > UAS-Imd* female flies (*n* = 20–24). (*g,j*) Toll pathway was artificially activated in fat bodies of male or virgin female flies in an adult-specific way. (*h–i*) Courtship index and mating success of *C564-GAL4; tub-GAL80^TS^>UAS-Toll10B* male flies (*n* = 19–21). (*k,l*) Copulation latency and mating success of *C564-GAL4; tub-GAL80TS > UAS-Toll10B* female flies (*n* = 19–20). Dunn's test in (*b*), (*e*), (*h*) and (*k*), Fisher's test in (*c*), (*f*), (*i*) and (*l*). Copulation latency is measured in seconds; courtship indices and mating success are presented as percentage. A detailed description of the statistics employed can be found in the electronic supplementary material. (Online version in colour.)

We next inquired if a strong genetic induction of the immune system without the presence of bacteria would affect the pre-copulatory behaviours shown by flies. To trigger each of the *Drosophila* immune arms in a specific manner, we genetically activated the immune pathways in the fat body, the primary site of humoral immune response, using the GAL4/UAS system [33]. To achieve this, we overexpressed Imd [34] or Toll^10B^ [35,36] using C564-GAL4 that targets the fat body and a few other tissues (e.g. gut and reproductive tract [37]). We found that constitutive activation of the Imd pathway does not significantly alter the courtship index or mating success in *C564 > Imd* males (figure 4*a–c*). Similarly, the activation of the Imd pathway did not influence female copulation latency or mating success (figure 4*d–f*).

We next tested the effects of activation of the Toll pathway. Since the expression of Toll^10B^ inhibits growth and causes developmental defects [38], we expressed Toll10B in an adult-specific way. We combined C564-GAL4 with the temperature-sensitive tub-GAL80^ts^ (GAL80^ts^) [39], an inhibitor of GAL4. At high temperature, GAL80 ceases to suppress GAL4, thereby allowing the expression of Toll^10B^. Intriguingly, we found that adult-specific activation of Toll^10B^ using the TARGET system did not affect the courtship index or mating success in *C564-GAL4; tub-GAL80^ts^>UAS-Toll^10B^* males when compared to controls (figure 4*g–i*). Furthermore, female sexual behaviour was unaffected in *C564-GAL4; tub-GAL80^ts^>UAS-Toll^10B^ females* (figure 4*j–l*)*.* These results, together with our previous findings, demonstrate that the activation of the immune system does not affect pre-copulatory behaviours in *Drosophila*.

## 4. Discussion

Reproduction and immunity are intricately linked traits central to an animal's fitness [1]. The factors that determine the trade-off between these energetically expensive traits remain poorly understood [1]. Here, we carried out detailed analyses of the effect of bacterial infections on pre-copulatory behaviours in *D. melanogaster*. We systematically tested the behavioural impact of infections with pathogenic (*S. marcescens, S. aureus* and *L. monocytogenes*) and non-pathogenic species (*Erwinia carotovora carotovora 15, E. coli* and *M. luteus*), using different bacterial doses, infection time points, and fly social contexts.

Our findings show that males infected with these diverse bacterial strains show normal levels of courtship intensity and mating success, even when presented with unfavourable targets. Further, females subjected to the same bacterial infections display high sexual receptivity and mating success. Consistent with our study, Keesey *et al.* [19] showed that infection with the lethal pathogen *Pseudomonas entomophila* leads to a small decrease in mating success in flies. Crucially, we report that generalized, constitutive and strong activation of the immune pathways by genetic means does not influence male or female pre-copulatory behaviours. These observations suggest that the preservation of pre-copulatory behaviours upon bacterial infection is not strain or doses specific but rather a general response in flies.

To optimize their fitness, animals are likely to avoid mating with partners infected with pathogens [40,41]. Birds and rodents have shown to discriminate against infected mates [40]. For example, greenfinches with a coccidian infection display reduced plumage coloration, reducing their chances of being selected as mates [42]. By contrast, our study shows that healthy and infected flies with *S. marcescens* and *S. aureus* pathogenic strains are equally chosen as patterns in competitive mating assays.

Importantly, our finding that *Drosophila* pre-copulatory behaviours are preserved during infections has parallels in other species. For instance, frogs infected with a deadly pathogen have comparable calling properties to that of uninfected individuals [43]. In addition, the immune activation by LPS injection in male crickets *Teleogryllus commodus* or *Gryllodes sigillatus* does not impact pre-copulatory traits [44,45]. To maintain pre-copulatory traits intact through the infection, immune-challenged flies may invest more of their available resources in reproduction, making a terminal investment [46]. However, while we observed a preservation of pre-copulatory behaviours, we did not find an increased expression of these reproductive traits.

Different reproductive traits may vary in their response to infections. For instance, *Drosophila* mated females reduce the rate of egg-laying in response to *E. coli* infection or exposure to LPS, likely decreasing the infection risk of their progeny [18]. *Drosophila* males infected with *P. aeruginosa*, display a decrease in sperm viability [47]. A reduced investment in post-copulatory traits but not in the execution of courtship behaviours could be explained by differential energetic costs associated with these traits. Post-copulatory traits, like sperm viability, ovulation and oviposition, might be energetically more expensive and therefore be under stronger selection pressure.

Several of the treatments employed in this study, such as *L. monocytogenes* or *E. coli* infections and genetic activation of the Toll pathway in the fat body, have been shown to interfere with the insulin signalling pathway and subsequently decrease nutrient storage or growth [27,38,48]. Yet, despite the marked metabolic switch triggered during systemic infections, flies retain their pre-copulatory behaviours. These findings highlight the relevance of reproductive behaviours and raise the question as to what mechanisms are in place to preserve them. Are the neuronal clusters or tissues dedicated to courtship insensitive to the systemic transformations triggered by infection? Conversely, are there mechanisms in place that actively maintain behavioural performance in response to infection-induced signals? The molecular and cellular machineries that control pre-copulatory behaviours upon bacteria detection remain to be determined.

It is important to highlight that systemic infections affect several non-reproductive behaviours in *Drosophila.* For instance, upon ingesting food contaminated with bacteria, flies reduce their activity and avoid harmed food via conditioned taste aversion mechanisms [49]. Moreover, upon contacting chemicals that normally activate the immune system, flies increase hygienic grooming [50]. Further, there is an interplay between immune activation and locomotion, with a subsequent impact on sleep, which depends on the pathogen type, the context and life history of the host [51]. Recently, it was reported that the Toll pathway in the fat body mediates a decrease in sleep in males infected with *M. luteus* [52]. By contrast, flies infected with *S. pneumoniae* show normal levels of activity but display altered sleep architecture and circadian rhythms [53].

In conclusion, despite the profound impact of bacterial infections on numerous metabolic, physiological and behavioural traits in *Drosophila*, pre-copulatory behaviours remain preserved, even in the face of deadly pathogens. Future experiments will investigate the mechanisms and the evolutionary ramifications of such strategy prioritization.

## Data Availability

The raw data and R code used for analysis has been deposited at Dryad: https://doi.org/10.5061/dryad.76hdr7szw [54]. The data are provided in the electronic supplementary material [55].
